# Capsular Polysaccharide Interferes with Biofilm Formation by *Pasteurella multocida* Serogroup A

**DOI:** 10.1128/mBio.01843-17

**Published:** 2017-11-21

**Authors:** Briana Petruzzi, Robert E. Briggs, W. Edward Swords, Cristina De Castro, Antonio Molinaro, Thomas J. Inzana

**Affiliations:** a Department of Biomedical Sciences and Pathobiology, Virginia-Maryland College of Veterinary Medicine, Virginia Tech, Blacksburg, Virginia, USA; b National Animal Disease Center, Agricultural Research Service, U.S. Department of Agriculture, Ames, Iowa, USA; c Department of Microbiology and Immunology, Wake Forest School of Medicine, Wake Forest University, Winston-Salem, North Carolina, USA; d Department of Agriculture, Università di Napoli Federico II, Naples, Italy; e Department of Chemical Sciences, Università di Napoli Federico II, Naples, Italy; f Virginia Tech Carilion School of Medicine, Roanoke, Virginia, USA; Ohio State University

**Keywords:** *Pasteurella multocida*, biofilms, capsule, chronic infection, exopolysaccharide

## Abstract

*Pasteurella multocida* is an important multihost animal and zoonotic pathogen that is capable of causing respiratory and multisystemic diseases, bacteremia, and bite wound infections. The glycosaminoglycan capsule of *P. multocida* is an essential virulence factor that protects the bacterium from host defenses. However, chronic infections (such as swine atrophic rhinitis and the carrier state in birds and other animals) may be associated with biofilm formation, which has not been characterized in *P. multocida*. Biofilm formation by clinical isolates was inversely related to capsule production and was confirmed with capsule-deficient mutants of highly encapsulated strains. Capsule-deficient mutants formed biofilms with a larger biomass that was thicker and smoother than the biofilm of encapsulated strains. Passage of a highly encapsulated, poor-biofilm-forming strain under conditions that favored biofilm formation resulted in the production of less capsular polysaccharide and a more robust biofilm, as did addition of hyaluronidase to the growth medium of all of the strains tested. The matrix material of the biofilm was composed predominately of a glycogen exopolysaccharide (EPS), as determined by gas chromatography-mass spectrometry, nuclear magnetic resonance, and enzymatic digestion. However, a putative glycogen synthesis locus was not differentially regulated when the bacteria were grown as a biofilm or planktonically, as determined by quantitative reverse transcriptase PCR. Therefore, the negatively charged capsule may interfere with biofilm formation by blocking adherence to a surface or by preventing the EPS matrix from encasing large numbers of bacterial cells. This is the first detailed description of biofilm formation and a glycogen EPS by *P. multocida*.

## IMPORTANCE

*Pasteurella multocida* is an important pathogen responsible for severe infections in food animals, domestic and wild birds, pet animals, and humans. *P. multocida* was first isolated by Louis Pasteur in 1880 and has been studied for over 130 years. However, aspects of its lifecycle have remained unknown. Although formation of a biofilm by *P. multocida* has been proposed, this report is the first to characterize biofilm formation by *P. multocida*. Of particular interest is that the biofilm matrix material contained a newly reported amylose-like glycogen as the exopolysaccharide component and that production of capsular polysaccharide (CPS) was inversely related to biofilm formation. However, even highly mucoid, poor-biofilm-forming strains could form abundant biofilms by loss of CPS or following *in vitro* passage under biofilm growth conditions. Therefore, the carrier state or subclinical chronic infections with *P. multocida* may result from CPS downregulation with concomitant enhanced biofilm formation.

## INTRODUCTION

*Pasteurella multocida* is a zoonotic (1), Gram-negative bacterium in the family *Pasteurellaceae*. *P. multocida* is part of the normal microbial flora of the upper respiratory tract of many animal species but is also a potential pathogen of many domestic and agriculturally important animals, such as dogs, cats, cattle, pigs, and avian species (2). *P. multocida* is also an important human pathogen following direct inoculation into subcutaneous tissues (e.g., bite wounds) (3). In hosts in which the innate immune response is compromised (such as prior viral infection, immunosuppression, stress, etc.) *P. multocida* is able to gain access to the lower respiratory tract and cause respiratory disease and systemic infection. In swine, *P. multocida* can cause a chronic polymicrobial infection (usually with *Bordetella bronchiseptica*) called atrophic rhinitis (4–6). However, *P. multocida* is not considered part of the normal flora of birds, in which it can be a highly invasive primary pathogen (7). Nonetheless, birds that recover from infection and obtain specific immunity can remain colonized by *P. multocida*, resulting in asymptomatic carriage and spread of the organism to nonimmune birds (8–10). Furthermore, birds can also become colonized with low-virulence *P. multocida* strains (11, 12). An important question is whether low-virulence strains can revert to a highly virulent phenotype if they infect naive animals. An essential virulence factor of *P. multocida* is a glycosaminoglycan capsular polysaccharide (CPS) that helps shield other surface antigens from the host immune system (13) and prevent phagocytosis and bactericidal activity, among other roles (14). There are five *P. multocida* CPS serogroups based on capsular antigens of distinct structural and antigenic specificity, designated A (15), B, D, E (16), and F (17). CPS serogroup A is composed of hyaluronic acid, serogroup D is a polysaccharide susceptible to enzymes that degrade chondroitin sulfates A and C and heparinase, and serogroup F is a polysaccharide similar to chondroitin (18). The serogroup B CPS is composed predominately of mannose but also contains arabinose and galactose, while the composition of the CPS of serogroup E strains has not been determined (14).

One of the most economically important diseases of cattle in the U.S. beef and dairy industries is bovine respiratory disease (BRD) (19). The cost of BRD to the cattle industry has been estimated at more than $500 million/year (20). The most common bacterial agents responsible for BRD include *Mannheimia haemolytica*, *Pasteurella multocida* (CPS serogroup A), *Histophilus somni*, and *Mycoplasma* spp. Isolation of more than one causative agent from a BRD infection is common (21). For example, *P. multocida* has been isolated from calves with lower respiratory tract disease following challenge or natural infection with *H. somni* (22, 23). Stresses such as crowding (feedlots), shipping, weaning, and viral infection further predispose the animals to infection (24). Transmission of BRD disease agents likely occurs by aerosol or physical contact between animals. Another common disease associated with *P. multocida*, as described above, is avian cholera, which can affect most avian species and occurs worldwide. However, some birds, such as turkeys and waterfowl (6, 10), are more susceptible to serious disease. The fatal infectious dose of *P. multocida* for mallards is as few as 12 cells (25). *P. multocida* can be transmitted through watering systems (26) (such as troughs and ponds that are shared by infected and healthy birds), by rodent infestations (27), and by the fecal-oral route (28), resulting in widespread infection and death. The most devastating outbreaks of avian cholera occur in locations where flocks of geese tend to migrate. For example, a single outbreak affected close to 20,000 birds and was associated with healthy migrating geese carrying *P. multocida* A:1 in nasal, oral, and cloacal samples (10). In humans, about 300,000 visits to emergency rooms are due to animal bite or scratch wounds (29), and *Pasteurella* spp. are isolated from ~50% of dog bites and ~75% of cat bites (30). However, bacteremia and systemic diseases without invasive animal contact have also been reported in humans (31).

*P. multocida* isolates from BRD and avian cholera (both serogroup A) have been reported to form a biofilm *in vitro* (32), and it has been proposed that swine atrophic rhinitis (serogroup D isolates) is a biofilm infection (33). However, characterization and careful analysis of a *P. multocida* biofilm and the biofilm matrix have not been reported. Biofilm infections within the host are a complex mixture of bacterial and host cells, exopolysaccharide (EPS), extracellular nucleic acids, trapped nutrients in water, and proteins. These bacterial communities are comparable to tissues formed by multicellular eukaryotes—the bacterial cells show cooperation, fluids and nutrients are circulated, and the bacteria are protected from unfavorable conditions in the external environment (34).

In this study, the formation of biofilm *in vitro* and the biofilm extracellular matrix of *P. multocida* serogroup A laboratory strains and clinical isolates was more thoroughly characterized. Of significance was determining that the amount of CPS produced by *P. multocida* is inversely proportional to the amount of biofilm formed and that highly encapsulated, poor-biofilm-forming strains can be converted to robust biofilm formers following loss or reduction of CPS by mutagenesis or *in vitro* passage. The biofilm matrix consisted of at least protein and a newly identified glycogen EPS. A putative polysaccharide synthesis and export locus was also identified but appeared to be constitutively expressed during biofilm formation or during planktonic growth.

## RESULTS

### Relationship between CPS production and biofilm formation by *P. multocida*.

**FIG 1:**
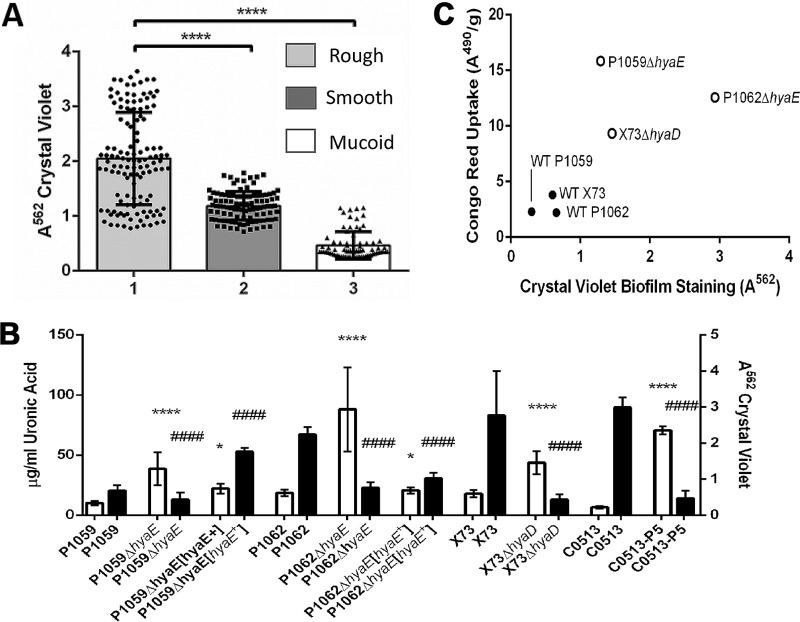
Correlation between CPS production and biofilm formation by *P. multocida* clinical isolates and laboratory strains. (A) Clinical and laboratory isolates were grouped on the basis of their colonial morphology (amount or lack of mucoid appearance and iridescence) on blood agar in relation to biofilm formation. The *y* axis represents the *A*_562_ after staining of the biofilm with CV and solubilization of the residue with 95% ethanol. Groups: 1, rough colonies/unencapsulated; 2, smooth colonies/intermediate encapsulation; 3, mucoid colonies/highly encapsulated. The amount of biofilm formed (as indicated by CV assay) by isolates in groups 2 and 3 was significantly smaller than the amount of biofilm formed by group 1 isolates (****, *P* ≤ 0.0001). (B) Comparison of biofilm formation by WT strains and the respective isogenic capsule-deficient mutants or an *in vitro*-passaged variant of WT C0153 (C0153-P5). The amounts of biofilm and CPS were determined by CV assay and uronic acid assay, respectively. The WT strains and the respective capsule-deficient mutants are listed on the *x* axis. The left *y* axis represents the concentration (μg/ml) of the uronic acid removed from the cell surface. The right *y* axis is the absorbance of solubilized CV after staining. White bars indicate the absorbance value from CV staining; black bars indicate uronic acid content. Biofilm formation was significantly higher in isolates producing less CPS. Significant differences between the parent and mutant strains in the CV assay are indicated by asterisks, and those in the uronic acid assay are indicated by number signs as follows: *, *P* ≤ 0.05; **** or ####, *P* ≤ 0.0001. (C) Correlation plot of *P. multocida* CR uptake absorbance values (*y* axis) and CV absorbance values for biofilms (*x* axis). The Pearson correlation coefficient is 0.7324 for all values and 0.9635 if P1059Δ*hyaE* is excluded. Encapsulated isolates are represented by solid dots, while acapsular isolates are represented by hollow dots.

A collection of clinical isolates and laboratory strains (see Table S1 in the supplemental material) of *P. multocida* was screened for the ability to form a biofilm by crystal violet (CV) assay. Upon initial screening, it was noted that there was an inverse correlation between the mucoid appearance of the colonies (an indication of the degree of encapsulation) and the amount of biofilm formed (Fig. 1A). To confirm the association between encapsulation and biofilm formation, we made isogenic mutants of the wild-type (WT) P1059 and P1062 strains by mutating the *hyaE* gene and of strain X73 by mutating *hyaD*. Recent clinical isolate C0153 was subcultured *in vitro* daily for five passages, by which time it was able to form a prominent biofilm. The *P. multocida* serogroup A capsule is composed of hyaluronic acid, which is not immunogenic because of its presence in host connective, epithelial, and neural tissues, thus negating the use of assays that utilize antibodies for antigen quantification. Therefore, to quantify CPS on the mutants, mid-log-phase cultures (optical density at 562 nm [OD_562_] of 0.6) were treated with hyaluronidase to release from the CPS the uronic acid, which was quantified by chemical assay. We confirmed the phenotypic observations (colony iridescence under incandescent lighting) that mutants P1062Δ*hyaE* and X73Δ*hyaD* and the passaged variant of C0153 all made significantly less CPS than the WT strains. WT P1062 produced 67.01 μg/ml uronic acid whereas P1062Δ*hyaE* produced 22.62 μg/ml uronic acid; WT X73 produced 83.02 μg/ml uronic acid, whereas X73Δ*hyaD* produced 15.90 μg/ml uronic acid (*P* < 0.0001 for both strains). *P. multocida* P1059Δ*hyaE* also made less uronic acid than WT P1059, but the difference was not significant (*P* = 0.1147) because WT P1059 produced relatively little uronic acid (CPS) in comparison with the other strains (20.39 μg/ml uronic acid for the parent versus 13.22 μg/ml uronic acid for P1059Δ*hyaE*) (Fig. 1B). Thus, all mutants with the same mutation in *hyaE* and the passaged strain were capsule deficient. To further confirm that a deficiency in CPS production was responsible for the enhanced biofilm formation seen, the mutation in strains P1059Δ*hyaE* and P1062Δ*hyaE* was complemented in *trans*. Uronic acid production was restored and enhanced in complemented mutant P1059Δ*hyaE*[*hyaE*], and biofilm formation was reduced. Biofilm formation by complemented mutant P1062Δ*hyaE*[*hyaE*] was also reduced to the same amount as the parent (Fig. 1B). WT *P. multocida* clinical isolate C0513 was a mucoid, poor-biofilm-forming strain that was isolated from a calf experimentally challenged with *H. somni* (22). WT C0513 was subcultured under biofilm-favoring growth conditions (five subcultures in RPMI medium every 48 h) until the strain could form a significant biofilm compared to the parent (*P* < 0.0001). WT C0513 produced 89.38 μg/ml uronic acid, while the subcultured variant (C0513-P5) produced 13.87 μg/ml uronic acid (*P* < 0.0001) (Fig. 1B), further supporting the idea that CPS production is inversely correlated with biofilm formation.

10.1128/mBio.01843-17.2 TABLE S1  Laboratory strains and clinical isolates used in this study. The isolates included commonly used laboratory strains and recent clinical isolates. Download TABLE S1, PDF file, 0.03 MB. Copyright © 2017 Petruzzi et al. 2017 Petruzzi et al. This content is distributed under the terms of the Creative Commons Attribution 4.0 International license.

We also observed that when *P. multocida* was grown on dextrose starch agar (DSA) supplemented with Congo red (CR), the presence of CPS inhibited CR uptake, enabling us to develop an assay to semiquantify CPS. Strains deficient in biofilm formation had low CR absorption indexes, while proficient biofilm-forming strains had significantly higher CR absorption indexes (P1059Δ*hyaE*, *P* ≤ 0.0001; P1062Δ*hyaE*, *P* ≤ 0.05; X73Δ*hyaD*, *P* ≤ 0.001) than the respective parent strains. Differences in CPS quantity between the parent and mutant strains were greater in the CR assay than in the uronic acid assay. A correlation graph was generated to evaluate the effectiveness of this assay in predicting biofilm-forming potential (Fig. 1C). However, the correlation between CR absorption and CV biofilm quantification for P1059Δ*hyaE* was an outlier on the correlation graph. Upon further investigation, we determined that there was a modification in the lipooligosaccharide (LOS) electrophoretic profile of P1059Δ*hyaE* that was distinct from that of the parent and not present in the other mutant strains (data not shown). The LOS modification may have contributed to enhanced CR uptake, particularly if the surface becomes more hydrophobic (35). If P1059Δ*hyaE* was excluded from statistical analysis of the correlation graph, the Pearson *r* value was 0.9635, further supporting a strong inverse correlation between CPS and biofilm formation. The CR assay was repeated with each group of clinical isolates (Fig. 1A), and CR uptake correlated inversely with colony morphology (iridescence). Group 1 consisted of rough, capsule-deficient strains, group 2 consisted of smooth, moderately encapsulated strains, and group 3 consisted of mucoid, highly encapsulated strains. Biofilms were quantified by CV uptake, and CPS was quantified by inhibition of CR uptake. Comparative measurements of the amount of biofilm formed and the amount of CPS produced consistently indicated that when more CPS was present, less biofilm was formed. Group 1 isolates overall produced a more robust biofilm than either group 2 or group 3 isolates (*P* < 0.0001 for both groups) on the basis of CV quantification. Group 2 isolates produced some biofilm, which was more than that produced by the mucoid strains, but the difference was not significant (*P* > 0.05).

**FIG 2:**
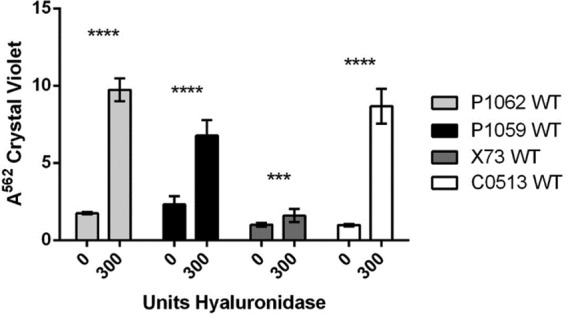
Effect of hyaluronidase on biofilm formation during growth. Either 0 or 300 μg of hyaluronidase was added to the culture medium of *P. multocida* strains under growth conditions favoring biofilm formation. After 2 days, the biofilms were rinsed and solubilized with 95% ethanol and the *A*^562^ values were determined. Significance values are based on comparison to the sample with no enzyme added. ***, *P* ≤ 0.001; ****, *P* ≤ 0.0001. White bars, WT C0513; dark gray bars, WT X73; black bars, WT P1059; light gray bars, WT P1062.

To further investigate the inverse relationship between CPS production and biofilm formation, hyaluronidase was added to the biofilm growth medium to degrade the CPS, as this process has been shown to at least partially eliminate the hyaluronic acid CPS from the surface of *P. multocida* serogroup A isolates (36, 37). When 300 U of hyaluronidase was added to encapsulated cultures of WT strains P1059, P1062, X73, and C0513, all of which were poor biofilm formers, biofilm formation by each strain increased significantly (*P* < 0.0001 for each strain), as determined by quantitative CV staining (Fig. 2). These collective results indicated that serogroup A CPS interfered with biofilm formation.

### Chemical and genomic analysis of the matrix EPS.

**FIG 3:**
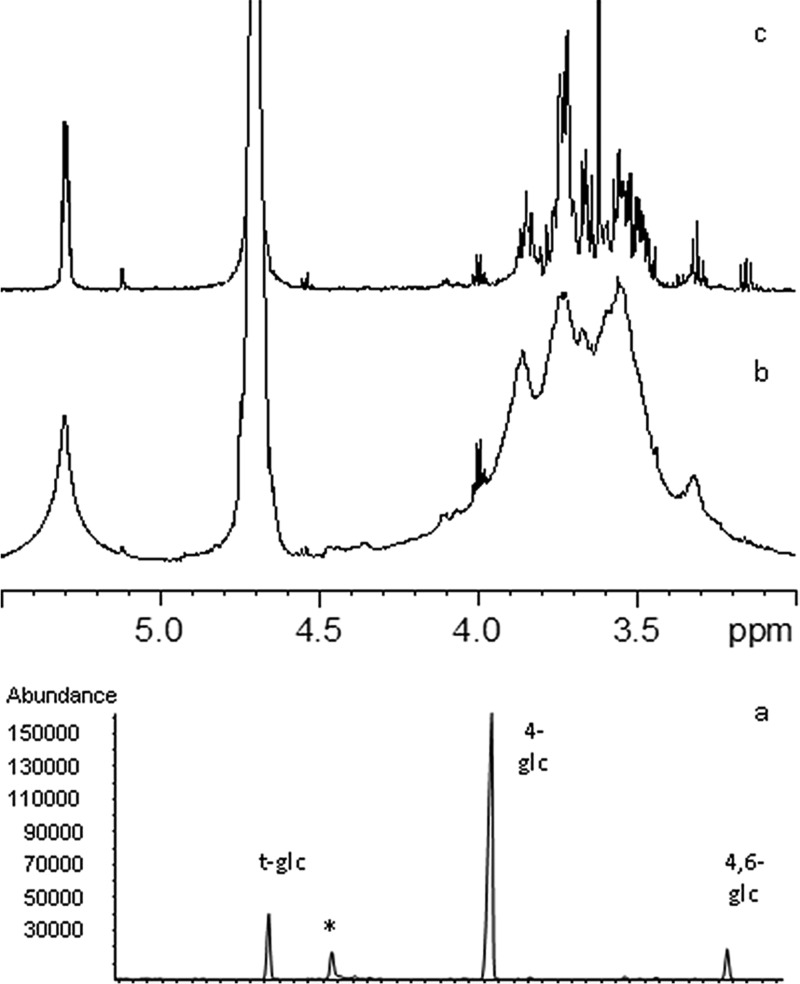
Structural analysis of the *P. multocida* serogroup A EPS. Panels: a, GC-MS chromatogram of partially methylated and acetylated alditols; b, proton spectrum of the intact EPS; c, proton spectrum of the EPS after overnight pullulanase digestion. *, impurity.

Gas chromatography-mass spectrometry (GC-MS) indicated that the EPS extracted from the biofilm matrix was a polymer composed of glucose that was terminally 4 or 4,6 linked (Fig. 3a). The nuclear magnetic resonance (NMR) proton spectrum displayed one main anomeric signal at 5.3 ppm (Fig. 3b) that is distinct for a glucose with an α configuration at the anomeric center. This information, along with chemical data, suggested that the EPS had a glycogen-like structure, later confirmed by digesting the polysaccharide with pullulanase, which depolymerized the polymer, as determined by NMR measurement (Fig. 3c).

A comparative BLAST search of available genome databases identified a putative glycogen synthesis locus in the *P. multocida* genome (Table 1). This locus consisted of six open reading frames that may encode the putative enzymes glucanotransferase (*malQ*), glucan branching protein (*glgB*), debranching protein (*glgX*), adenylyltransferase (*glgC*), synthase (*glgA*), and phosphorylase (*glgP*). The encoded proteins shared the greatest identity with those of other members of the family *Pasteurellaceae*, most notably *Aggregatibacter* spp., *Necropsobacter rosorum*, and *Haemophilus influenzae* (Table 1).

**TABLE 1:** *P. multocida* WT strain P1059 genes with amino acid identity to glycogen synthesis proteins in related bacteria

Locus tag	Gene	Putative gene product	% Amino acid sequence identity (bacterium)^a^	Gene size (bp)
PM_RS02805	*malQ*	4-α-Glucanotransferase	66 (*Aggregatibacter* *segnis*), 64 (*Aggregatibacter* *aphrophilus*)	2,094
PM_RS02810	*glgB*	1,4-α-Glucan-branching enzyme	87 (*A. segnis*), 86 (*Haemophilus felis*)	2,193
PM_RS02815	*glgX*	Glycogen debranching protein	69 (*H. felis*), 67 (*A. aphrophilus*)	2,019
PM_RS02820	*glgC*	Glucose-1-phosphate adenyltransferase	94 (*H. felis*), 91 (*Necropsobacter rosorum*)	1,308
PM_RS02825	*glgA*	Glycogen synthase	86 (*H. felis*), 84 (*A. segnis*)	1,443
PM_RS02830	*glgP*	Glycogen phosphorylase	89 (*H. felis*), 82 (*A. aphrophilus*)	2,457

^a^ Genera and species other than *Pasteurella* are listed.

### Enzymatic treatment of biofilms.

**FIG 4:**
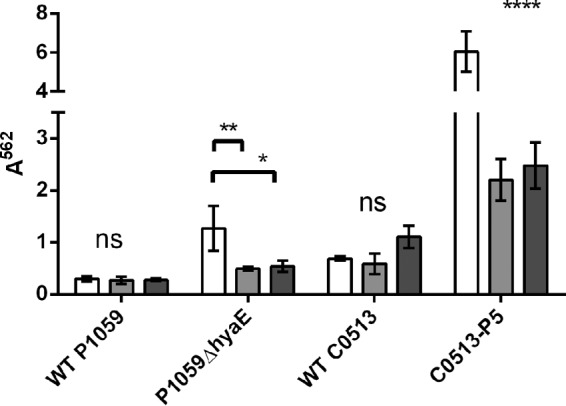
Enzyme digestion of biofilm matrix. Prior to inoculation of the culture medium with *P. multocida*, 0.1 mg/ml α-amylase or proteinase K was added; this was followed by incubation for 48 h. Biofilms were rinsed, stained with CV, and solubilized with ethanol, and the *A*^562^ was determined. White bars, no treatment; light gray bars, treatment with proteinase K; dark gray bars, treatment with α-amylase. Significance values are based on comparison to the sample with no enzyme added. *, *P* ≤ 0.05; **, *P* ≤ 0.01; ****, *P* ≤ 0.0001; ns, not significant.

Developing biofilms were treated with proteinase K and α-amylase to determine their effect on biofilm development and to evaluate matrix composition (Fig. 4). The WT P1059 biofilm retained 91 and 93% of the CV stain after exposure to proteinase K (*P* = 0.9957) and α-amylase (*P* = 0.9977), respectively, compared to biofilm growth in the absence of these enzymes. These results reflect the lack of biofilm formed by WT strain P1059. In the more robust biofilm-forming, CPS-deficient strain P1059Δ*hyaE*, there was 61 and 57% less biofilm after growth with proteinase K and α-amylase, respectively (*P* = 0.0065 and *P* = 0.0118, respectively). Therefore, while more than half of the biofilm matrix was removed after enzymatic treatment of P1059Δ*hyaE*, less than 10% of the WT P1059 matrix was removed after treatment with either enzyme. Similar results were obtained with highly encapsulated, biofilm-poor WT strain C0513 and the *in vitro*-passaged variant C0513-P5. In WT C0513, 79% of the biofilm CV stain was retained after proteinase K treatment (*P* = 0.9123) and a small but insignificant increase in CV staining occurred after treatment with α-amylase (*P* = 0.2249). In subcultured variant C0513-P5, 44% of the biofilm CV stain was lost after proteinase K treatment (*P* < 0.0001) and 37% of the biofilm CV stain was lost after α-amylase treatment (*P* < 0.0001). The *H. somni* 2336 biofilm was not affected by treatment with α-amylase but was diminished to a similar extent after treatment with proteinase K (data not shown).

### SEM.

**FIG 5:**
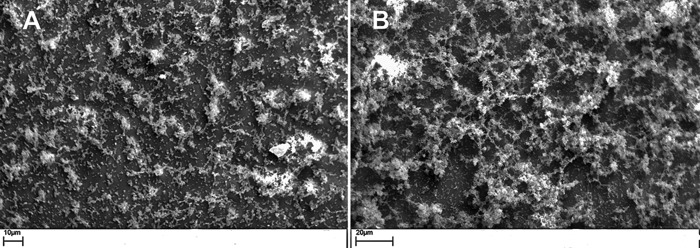
SEM images of *P. multocida* biofilms after 48 h of incubation on glass coverslips. (A) WT strain P1059. (B) P1059Δ*hyaE* biofilm formation.

WT P1059 and P1059Δ*hyaE* biofilms were grown on abiotic coverslips and examined for formation and structure by scanning electron microscopy (SEM). WT P1059 formed microcolonies surrounded by small amounts of biofilm matrix. Individual bacterial cells or small clusters of two to six bacteria adhered to the glass coverslip and were dispersed between microcolonies (Fig. 5A). The P1059Δ*hyaE* biofilm matrix was clearly visible, included peaks and valleys characteristic of biofilm matrices, and was more extensive than the biofilm of the parent. Some individual bacterial cells adhered to the glass coverslip below the matrix, similar to the WT (Fig. 5B).

### CLSM.

**FIG 6:**
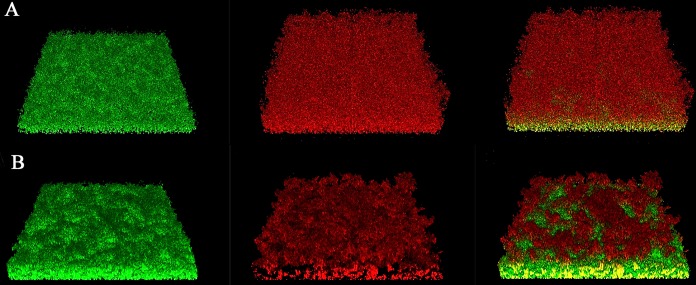
CSLM z-stack showing live/dead staining of WT P1059 during biofilm formation. (A) WT P1059. (B) P1059Δ*hyaE*. Live staining, left column; dead staining, center column; live-dead overlay, right column.

Two-day-old biofilms of WT P1059 and P1059Δ*hyaE* were imaged by confocal laser scanning microscopy (CLSM), converted into z-stacks, and analyzed for structural characteristics (Table 2). Biofilms of WT P1059 (*n* = 10) were best characterized as a cell monolayer with an average thickness of 0.001719 µm^3^/µm^2^ and a roughness coefficient of 2, which indicated maximum roughness. Of interest, live/dead staining of the WT P1059 monolayer identified living cells adhering to the glass coverslip, while dead cell debris was present on the biofilm exterior (Fig. 6A; Fig. S1A). The P1059Δ*hyaE* biofilm had an average biomass volume of 21 µm^3^/µm^2^, and live/dead staining indicated that the biofilm was composed primarily of living cells. The topmost layer appeared to have small clusters of dead cellular debris (Fig. 6B; Fig. S1B).

10.1128/mBio.01843-17.1 FIG S1  Cross sections of biofilms of WT P1059 (A) and P1059Δ*hyaE* (B) by CLSM. This is a cross-sectional view through the center of the biofilm shown in Fig. 6. The biofilm was stained with SYTO 9. The left column and top row show the height and thickness of each biofilm, which is larger and thicker for capsule-deficient mutant P1059Δ*hyaE* than for the parent strain. Download FIG S1, PDF file, 0.1 MB. Copyright © 2017 Petruzzi et al. 2017 Petruzzi et al. This content is distributed under the terms of the Creative Commons Attribution 4.0 International license.

**TABLE 2:** Comstat analysis results obtained from CSLM z-stack images

Strain^a^	Avg biomass (µm^3^/µm^2^) ± SD	Avg thickness (µm) ± SD	Max thickness (µm)	Roughness coefficient (0–2)	Surface area/biovolume ratio
WT P1059	0.0449 ± 0.0032	0.001719 ± 0.0003	4.5	2	9.92
P1059Δ*hyaE*	21 ± 3.23	29.79 ± 4.73	34.31	1	5.05

^a^ *n =* 10 for both strains.

### qRT-PCR of putative EPS matrix genes.

**FIG 7:**
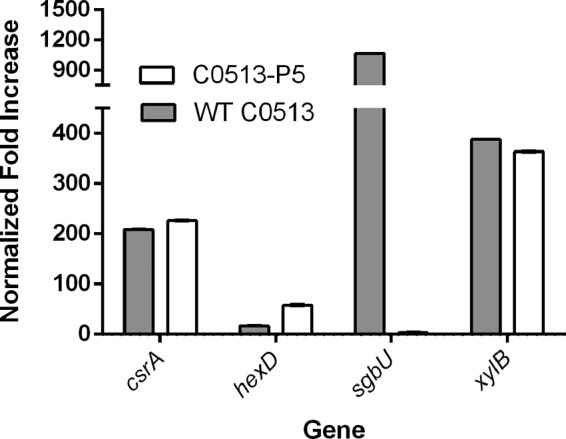
Normalized fold increases in genes significantly upregulated during biofilm formation. WT strain C0513 was grown for 48 h under stationary conditions to promote biofilm growth and subcultured five consecutive times under the same growth conditions to obtain strain C0153-P5. Gray bars, WT strain C0513 (biofilm-deficient strain); white bars, variant C0513-P5 (biofilm-proficient strain).

The expression of genes proposed to encode the glucan EPS of subcultured variant C0513-P5 and WT parent C0513 during planktonic growth was similar, with only minor differences noted on the basis of quantitative reverse transcriptase PCR (qRT-PCR). Gene expression of both WT C0513 and C0513-P5 during planktonic growth was also compared with gene expression of the same strains during biofilm formation (Fig. 7). Constitutively expressed *gyrB* was used as a control to normalize the fold differences between strains. Most of the genes in the CPS locus and the putative glycogen synthesis locus, genes encoding putative glycosyltransferases (38, 39), genes involved in lipopolysaccharide synthesis (e.g., *waaA* [40]), and genes that encode other putative EPS biosynthesis proteins (e.g., *opsX* [41]) were not differentially expressed between cells of either strain grown planktonically or as a biofilm (not shown). Therefore, differences in gene expression that would distinguish a proficient biofilm former from a deficient one were not identified in those genes. However, the genes for CsrA (carbon storage regulator A), HexD (CPS export), and XylB (xylulose kinase) were upregulated in both the parent and the proficient biofilm former C0513-P5. However, of interest was that the expression of SgbU (l-xyulose-5-phosphate 3-epimerase) was greatly enhanced in only WT parent strain C0513.

## DISCUSSION

*P. multocida* can cause acute or chronic infections in many animal species and humans. Chronic infections include atrophic rhinitis in swine and snuffles in rabbits, and birds and cattle may carry *P. multocida* asymptomatically and act as host carriers. Avian *P. multocida* carrier animals demonstrate few or no outward signs of infection (42). Carrier migratory birds, such as geese (9), may act as persistent reservoirs of infection that can spread virulent *P. multocida* as they migrate between water supplies (43, 44). Samuel et al. (12) reported that a parenteral challenge of mallards with “relatively low-virulence” *P. multocida* resulted in the establishment of a carrier state in both challenged birds and birds in contact with the infected birds. Of interest is that Pritchett et al. (45) described endemic fowl cholera in flocks of leghorns from which isolates that formed “blue” colonies (acapsular phenotype) were associated with a relatively high incidence of the carrier state in birds, relatively little disease, and a high incidence of contact carrier birds. Subsequently, Pritchett et al. (11) reported that an intranasal challenge of leghorns with “fluorescent colonies” (presumably encapsulated) of an epidemic strain resulted in a high mortality rate but a low incidence of carrier animals and little spread to contact birds. In chronic or asymptomatic infections by “blue” colony types, the bacteria may be present as a biofilm in which the bacteria are relatively innocuous and there is little inflammatory response (7, 25, 46). However, bacteria shed from the biofilm may be in a planktonic state, which may be more pathogenic and contagious to susceptible animals. In this transmission model, biofilm formation is essential to the spread of potential pathogens. Although biofilm formation has been proposed as a component of some *P. multocida* infections (32, 33), this is the first study to more thoroughly characterize the composition and formation of *P. multocida* biofilms.

Examination of biofilms by a wide variety of clinical and laboratory isolates indicated that all of the strains of *P. multocida* could form some biofilm but that there was a wide variation in the amount of biofilm that individual strains could form. Only isolates of nontoxigenic CPS serogroups A and F of avian or bovine origin were reported. However, biofilm formation by serogroup D isolates of porcine origin was also confirmed in our assays (not shown), as proposed previously (33). When the extent of biofilm formation was compared with colony morphology under iridescent light, it was noted that there was an inverse relationship between the iridescence and how mucoid the colonies were and the amount of biofilm formed by that strain. It has been clearly established that cells with the most CPS on their surface form the most mucoid, iridescent colonies, with smooth colonies containing less CPS and smaller blue colonies (rough) being CPS deficient (47, 48). Inverse correlations between CPS content and biofilm formation that are similar to the results described here for *P. multocida* have also been reported for *Neisseria meningitidis* (49), *Escherichia coli* (50, 51), and other bacterial species (49, 52).

To confirm the inverse relationship between CPS content and biofilm content, an accurate assay to quantify CPS was necessary. Immunoassays are unavailable because antibodies against the type A CPS hyaluronic acid are difficult to obtain because this CPS is recognized as “self” by the immune system. An alternative method to quantify *P. multocida* type A CPS production is by observing the colony morphology on solid agar medium (47). This method has been used to distinguish encapsulated, virulent *P. multocida* from less virulent or avirulent decapsulated *P. multocida* for almost a century (53). However, this method is not quantitative and we determined that *P. multocida* is capable of producing more than one polysaccharide, prompting the need for a quantification method that can differentiate hyaluronic acid from glycogen and potentially xylan (54). Several methods that have been used to quantify hyaluronic acid were not useful in distinguishing hyaluronic acid from other or similar polysaccharides because of a lack of test specificity; these assays use reagents that react with carbohydrates but not specifically hyaluronic acid (13, 55, 56). Therefore, enzymatic removal of the CPS from the cell surface was used to quantify the uronic acid present by using a carbazole assay (57). In addition, we determined that the CPS inhibits CR uptake by the bacteria because CR preferentially binds to neutral carbohydrates (58). Therefore, the amount of CPS produced was inversely proportional to CR uptake by *P. multocida* isolates. This assay correlated with biofilm formation based on larger index values of isolates that produced a proficient biofilm and a smaller index value that correlated with isolates that were deficient in biofilm formation.

Further evidence that CPS inhibited biofilm formation was supported through the use of three isogenic CPS-deficient mutants with mutations in *hyaE* or *hyaD*. Subculture of *P. multocida in vitro* has long been known to result in spontaneous loss of CPS (48). We confirmed this observation by growing an encapsulated isolate under biofilm-favoring growth conditions for five passages, resulting in reduced CPS production while simultaneously increasing biofilm formation (strain C0513-P5). Thus, the removal of CPS (based on uronic acid assay of enzymatically treated cells and the CR uptake assay) enhanced proficient biofilm formation in a previously poor-biofilm-forming strain. Therefore, CPS may inhibit biofilm formation by blocking surface proteins essential for adherence, the first step of biofilm formation (59). CPS has also been shown to physically block adhesion factors essential to biofilm formation in *E. coli* (60, 61). Complementation of mutants P1059Δ*hyaE* and P1062Δ*hyaE* with *hyaE* in *trans* reduced biofilm formation and enhanced uronic acid content in both complemented recombinant strains, further supporting the idea that the presence and the amount of CPS interfered with biofilm formation. Finally, hyaluronidase treatment to remove most of the CPS from the cell surface of each parent strain significantly enhanced biofilm formation but had little to no effect on rough, good-biofilm-forming strains or CPS-deficient strains (data for the latter not shown). However, hyaluronidase treatment was less effective at enhancing biofilm formation by WT X73 than that by the other three strains, though it was still significantly able to do so. Why hyaluronidase was not as effective in enhancing biofilm formation in strain X73 could be explained if this strain also produces the β-(1,4)-d-xylan polysaccharide that has been reported for at least one other strain (54). A d-xylan polysaccharide would not be susceptible to removal by hyaluronidase.

Biofilms are composed primarily of cells encased in a matrix material that is predominately EPS but also proteins and extracellular nucleic acids (34). Once it was established that some strains of *P. multocida* could form a substantial biofilm, we sought to determine if the matrix material consists of a polysaccharide distinct from the CPS. By using procedures used previously to isolate EPS from *H. somni* (62), an α-(1,4)-linked polymer of glucose (glycogen) was isolated. This is the first description of a glycogen polymer produced by *P. multocida*. However, qRT-PCR indicated that the putative genes responsible for glycogen synthesis were not upregulated during biofilm formation, indicating that some glycogen may always be produced (63), thus accounting for small amounts of biofilm in all of the strains tested. It is possible that the large amount of CPS produced by mucoid strains masked the presence of the glycogen. A wide variety of bacterial genera have the genes to make proteins to synthesize a glycogen polymer, and the genera with the greatest amino acid sequence identity to those in *P. multocida* are other members of the family *Pasteurellaceae*. Of interest is that some of these proteins are closest in identity to those from *Haemophilus felis*, which, like *P. multocida*, lives in the upper respiratory tracts of cats (64). Surface expression of CPS may also inhibit biofilm formation by blocking cell surface adhesins (59). The addition of hyaluronic acid, the component of serogroup A CPS, to the biofilm growth medium had no effect on biofilm formation, likely because the added polysaccharide was not associated with the cell surface, as it is when produced by the cell. However, as mentioned above, the addition of hyaluronidase to hydrolyze the serogroup A CPS present did enhance biofilm formation by highly encapsulated poor biofilm formers. Furthermore, at least one gene responsible for CPS export was upregulated during proficient biofilm formation, indicating that the EPS and CPD may be exported by a common pathway.

Enzymatic digestions of developing biofilms were used to determine the relative amounts of protein and carbohydrate within the biofilm. Addition of proteinase K and α-amylase enzymes to the biofilm growth medium resulted in partial digestion of the biofilm material but had little effect on isolates deficient in biofilm formation. These results supported the idea that the biofilm matrix consisted of glycogen, the substrate for α-amylase, and that the biofilm matrix was also composed of protein or that the matrix structure was dependent on a protein scaffolding.

Previous studies have shown that *P. multocida* can display substantial quantities of lipids on its surface, causing the bacterium to become hydrophobic (65, 66) and increasing the adherence of the bacterium to surfaces (66); an essential early step in biofilm formation. However, in studies not shown, there was no difference in hydrophobicity between the parent and acapsular mutant strains (as determined by the bacteria moving from an aqueous phase to an organic phase), indicating that hydrophobic interactions did not contribute to biofilm formation in *P. multocida*. Autoaggregation can also contribute to biofilm formation and is often facilitated by EPS and/or bacterial adhesins (67, 68). However, no differences in autoaggregation were noted between proficient and deficient biofilm-forming strains (*P* = 0.227) (data not shown), indicating that cell-to-cell interactions were not inhibited by CPS, and autoaggregation was not a good predictor of biofilm formation by *P. multocida*.

Changing growth conditions from those that favor planktonic growth (approximately 6 h with rapid shaking) to those that favor biofilm growth (>6 h under stationary conditions) was sufficient to initiate changes in gene expression. *H. somni* and *P. multocida* are genetically related. Therefore, homologues of genes shown to be upregulated during *H. somni* biofilm formation (69) were included in our qRT-PCR assays during biofilm formation by *P. multocida*. As expected, some of the genes upregulated during *H. somni* biofilm formation were also upregulated during *P. multocida* biofilm formation. For example, the gene for the carbon storage regulator CsrA was upregulated during biofilm formation by both *H. somni* (62) and *P. multocida*. However, CsrA is an RNA-binding protein that represses biofilm formation in some Gram-negative bacteria, such as *E. coli* (70) and *Campylobacter jejuni* (71). In *E. coli*, CsrA influences biofilm suppression and dispersal primarily through its regulatory effect on glycogen synthesis and catabolism (70). Although the effect of CsrA on biofilm formation is reversed between *E. coli* and *P. multocida*, upregulation of CsrA enhances glycogen synthesis, which in *P. multocida* enhances biofilm formation. Xylulose kinase (XylB) and l-xyulose-5-phosphate 3-epimerase (SgbU) were also upregulated during *P. multocida* biofilm formation (though in the latter only in the parent strain). A β(1-4)-d-xylan polysaccharide has been identified as an additional polysaccharide of at least one other *P. multocida* serogroup A strain (54). Therefore, xylan could be another EPS that forms the biofilm matrix of some strains, such as C0153, which was used for qRT-PCR, but not for EPS purification. Expression of the gene for SgbU was examined because it was also upregulated during *H. somni* biofilm formation (62). Of interest was that the gene for SgbU was upregulated only during biofilm formation by the parent and not during that by the mutant. Therefore, we suspect that SgbU was not directly related to biofilm formation but may be affected by a global regulator, such as Fis, which also downregulates CPS expression following subculture (71).

A gene in the *P. multocida* CPS locus, *hexD*, encodes a protein required for polysaccharide export (14, 15) and was upregulated during biofilm formation. Therefore, one or more genes responsible for CPS export may also contribute to EPS export. Genes involved in hyaluronic acid biosynthesis are in region 2 and were neither upregulated or downregulated. Genes in the putative glycogen synthesis locus were not differentially regulated during proficient biofilm formation versus deficient biofilm formation. Therefore, glycogen may be constitutively expressed but can only contribute to matrix formation when CPS is absent and adherence and biofilm formation can be initiated.

In summary, we have demonstrated that biofilm formation by *P. multocida in vitro* is inversely related to CPS production. The biofilm was composed of protein and a newly identified glycogen amylose EPS (and likely extracellular DNA). Since isolates that produce less CPS are less virulent and proficient biofilm formers, we propose that downregulation of CPS (72) may result in chronic pasteurellosis and avian carriers because of enhanced biofilm formation.

## MATERIALS AND METHODS

### Isolates and growth conditions.

The laboratory and clinical isolates of *P. multocida* used in this study and their sources are described in Table S1. Strain C0513 was isolated from the transtracheal wash of a calf following an experimental challenge (22) with *H. somni*. Prior to the challenge, nasopharyngeal cultures from the calf were negative for *P. multocida* and other respiratory pathogens. All *P. multocida* strains were cultured on brain heart infusion (BHI) or Columbia blood agar (BD, Franklin Lakes, NJ) supplemented with 5% defibrinated sheep blood (HemoStat Laboratories Inc., Dixon, CA) or on DSA with or without 0.005% CR supplementation. Agar-grown cultures were grown at 37°C with 6% CO_2_. Broth cultures were grown in BHI or RPMI 1640 medium without glutamine or phenol red (Lonza, Walkersville, MD). For biofilm formation, 50 μl of mid-log-phase *P. multocida* was inoculated into 5 ml of RPMI 1640 in a 50-ml polyethylene tube and incubated without shaking at 37°C in 6% CO_2_ for at least 48 h.

All *P. multocida* isolates were confirmed and serogrouped by multiplex PCR as described elsewhere (73). All of the isolates used in this study were nontoxigenic and either serogroup A or F or untypeable (not A, B, D, E, or F).

### Isolation of a biofilm-proficient *P. multocida* variant.

WT isolate C0513 was subcultured every 48 h under conditions favoring proficient biofilm growth (described above) until the isolate was capable of producing a proficient biofilm, which occurred after five subcultures. This variant is referred to as C0513-P5.

### Construction of acapsular *P. multocida* mutants.

Gene replacement mutants of WT *P. multocida* isolates WT P1059 (avian, serogroup A:3), WT P1062 (bovine, serogroup A:3), and WT X73 (avian, serogroup A:1) were generated by using previously described techniques, with minor modifications (74). Briefly, DNA fragments containing the *hyaE* gene of WT P1059 and WT P1062 were amplified with forward primer 5′ ATGAAAAAGGTTAATATCATTGG 3′ and reverse primer 5′ TTAACCTTGCTTGAATCGTTTACC 3′ to produce an approximately 1,870-bp fragment containing the *hyaE* coding region of each isolate. These PCR products were cloned into pCR2.1 (Invitrogen, La Jolla, CA) and amplified with deletion primers 5′ AAAGATATCTTGGTTTACTTCAATAATTTC 3′ and 5′ AAAGATATCACTGCATCTGTTCAATCAACGAGC 3′, which produced a linear product with pCR2.1 flanked by the upstream and downstream arms of the replacement plasmid. Digestion with EcoRV (sites contained in the primers) and ligation recircularized the plasmid, deleting amino acids 239 through 359 of the encoded protein and substituting isoleucine for the leucine residue at former position 360. An attempt was made to frameshift the deleted gene in WT P1062 by inserting a SmaI linker (5′ CCCCGGGG 3′) into the EcoRV site, but the product produced was found to contain a series of three of the linkers, which restored the reading frame to encode an additional eight amino acids (Pro Arg Gly Pro Gly Ala Pro Gly) in the deletion site.

A DNA fragment containing a portion of the *hyaE* and *hyaD* coding regions of WT X73 was amplified with forward primer 5′ GAAGATGCGCATGAAGCCAATCGCATT 3′ and reverse primer 5′ GCCATTTGGTTTAGACATGATG 3′ to produce an approximately 1,680-bp product. This PCR product was cloned into pCR2.1, digested with the BglII restriction endonuclease, and ligated to introduce a 225-bp deletion in the *hyaD* gene in frame into the coding region.

Each of the above DNA segments from WT strains P1059, P1062, and X73 replacement DNA segments were excised from pCR2.1, cloned into pGA301*ori*, introduced by electroporation into the respective strains, and then handled as previously described (74). PCR was used to confirm that kanamycin-sensitive colonies lacked plasmid pGA301*ori*. Mutations in the CPS biosynthetic locus were confirmed by PCR with the primers described above. P1059Δ*hyaE* and P1062Δ*hyaE* yielded a PCR product approximately 360 bp smaller than that of their WT counterparts, and the X-73Δ*hyaD* PCR product was approximately 220 bp smaller than that of its WT counterpart. Each mutant appeared noniridescent when grown on DSA under oblique illumination, whereas each parent was clearly iridescent because of capsule formation.

To complement the mutation in P1062Δ*hyaE*, a forward primer (5′ AAAAAGGATCCCAAGCGTTGGGTAAAAAAACCGCTTA 3′) and reverse primer (5′ AAAAGGATCCTCCATAGATTCCGCCGACTTTTCA 3′) were used to amplify the promoter and coding region of *hyaE* by using the WT P1062 genomic template. The BamHI-digested product was ligated into pBC SK− (Stratagene) containing a kanamycin resistance marker (pUC4K) in the SalI site. The backbone of the resulting plasmid was replaced by digestion with BssHII and ligated to a DNA fragment containing the *Mannheimia haemolytica* pD80 (a 4.2-kb ampicillin resistance plasmid) origin of replication. The resultant product was electroporated into competent WT P1062 as previously described (75). *P. multocida* was made competent by growth to mid-log phase, and 37 μl of hyaluronidase solution (10 mg/ml) was added to 10 ml of culture and incubated for 30 min without shaking. The bacteria were washed three times with ice-cold 10% glycerol, and the bacteria were resuspended in any solution remaining after the final wash. About 50 μl of cell suspension was transferred to cold electroporation cuvettes for electroporation into the mutant strains (74).

### RNA extraction, PCR, qRT-PCR, and BLAST analysis.

Primer set 1, specific for *toxA*, was used as described by Kamp et al. (76). All PCRs were performed in an Eppendorf Mastercycler pro PCR system with vapo.protect technology (Eppendorf, Germany) by using OneTaq reagents in accordance with the manufacturer’s instructions (New England Biolabs, Ipswich, MA).

At least three technical replicates of WT C0513 and C0513-P5 biofilm and planktonic cultures were pooled to obtain RNA from each growth phase. RNA was isolated with Qiagen RNAprotect bacterial reagent, Qiagen QiaShredder, and Qiagen RNeasy kits (Qiagen, Hilden, Germany) in accordance with the manufacturer’s instructions for prokaryotic RNA. RNA was transcribed into cDNA with the Quanta qScript kit (Quanta Biosciences, Gaithersburg, MD) in accordance with the manufacturer’s instructions. qRT-PCR was performed on an Applied Biosciences 7300 real-time PCR system (Applied Biosystems, Foster City, CA) with the Quanta SYBR FastMix kit (Quanta Biosciences, Gaithersburg, MD) in accordance with the manufacturer’s instructions. Quantitative real-time PCR was performed in triplicate for each primer set with two biological replicates of each strain. The primers used are described in Table S2. A primer set identifying *gyrB* was used as a control. The 2^−ΔΔ*CT*^ method (77) was used to calculate the normalized fold differences in gene expression. Genes analyzed by qRT-PCR were selected on the basis of their relevance to biofilm formation in the related species *H. somni* (62), homology to genes involved in glycogen synthesis (Table 1), or *P. multocida* CPS production (15).

10.1128/mBio.01843-17.3 TABLE S2  Primers used for qRT-PCR analysis in this study. Download TABLE S2, PDF file, 0.2 MB. Copyright © 2017 Petruzzi et al. 2017 Petruzzi et al. This content is distributed under the terms of the Creative Commons Attribution 4.0 International license.

Initially, the sequence of the gene for *E. coli* glycogen synthase (GlgA) (78) was used to determine that a *glgA* homologue and related genes in a glycogen locus exist in the *P. multocida* genome. A BLASTP search (79) was then used to identify the closest homologies to annotated genes of other genera.

### Biofilm quantification.

A modification of the CV staining method of Sandal et al. (69) was used to quantify biofilm formation. Two hundred microliters of 0.1% CV was gently added to a tube containing 5 ml of bacterial culture and incubated at room temperature for 10 to 15 min. The medium and CV were removed by pipetting, and the tube was gently washed with phosphate-buffered saline (PBS), pH 7.2. To quantify biofilm formation, the CV was solubilized with 500 μl of 95% ethanol, 200 μl was transferred to a 96-well microtiter plate, and the OD_562_ was determined with a VMax Kinetic Plate Reader (Molecular Devices, Sunnyvale, CA). At least three biofilms were tested for each biological replicate.

### Capsule quantification.

Isolates and strains were grown in BHI broth to mid-log phase, washed with saline, and resuspended to an OD_562_ of 0.7 in 10 ml of saline. Bacterial suspensions were incubated at 37°C with 200 μl of 5 mg/ml hyaluronidase for 30 min and then harvested at 10,000 × *g* for 10 min. A uronic acid assay of the supernatant was performed (57), and the results were quantified in comparison to purified uronic acid control standards. The OD_562_ was recorded with a VMax Kinetic Plate Reader (Molecular Devices). In addition, isolates were grown overnight on BHI agar supplemented with 0.005% CR. Bacterial colonies were removed from the agar surface and suspended in a preweighed 1.5-ml tube containing 1 ml of PBS. The tubes were centrifuged at 10,000 × *g* for 5 min, and the PBS was discarded. The wet weight of each sample was determined, and the bacteria were resuspended in 1 ml of 1% SDS in PBS, releasing the CR-bound material from the bacterial lysate. Two hundred microliters of each sample was transferred to a 96-well microtiter plate. The OD_490_ was determined with a VMax Kinetic Plate Reader (Molecular Devices). The CR absorption index (CRA) was calculated with the formula CRA = *A*^490^/(*W*_2_ − *W*_1_), where *W*_2_ is the weight of the tube and PBS buffer after the addition of bacteria and *W*_1_ is the weight of the tube and PBS buffer before the addition of bacteria.

### Purification of EPS from the biofilm.

Biofilm EPS was extracted from *P. multocida* as previously described (62), with RPMI 1640 without phenol red or glutamine (Lonza, Walkersville, MD) as the growth medium. After the biofilm matured, the growth medium was carefully removed, leaving the biofilm and sediment intact. The purified EPS was lyophilized.

### Chemical analysis of EPS.

EPS composition and substitution patterns were determined by analyzing monosaccharides as acetylated methylglycoside derivatives of the partially methylated and acetylated alditols (80). The conditions used for GC-MS were the same for each derivative, and it was performed on an Agilent 6850A gas chromatograph equipped with an SPB-5 capillary column (Supelco; 30 m by 0.25 mm [inside diameter]; flow rate of 0.8 ml min^−1^ with He as the gas carrier) interfaced with a 5973N mass detector. Electron impact mass spectra were recorded with an ionization energy of 70 eV and an ionizing current of 0.2 mA. The temperature program used was set at 150°C for 5 min, 3°C/min up to 280°C, and 300°C for 5 min. EPS glycogen was quantified by the anthrone reagent method (81).

Pullulanase (P5420; Sigma-Aldrich, Milan, Italy) digestion prior to NMR spectroscopy was carried out by adding 20 μl (2 U) of the suspension to the sample and then incubating it at 37°C overnight. Proton spectra were recorded in D_2_O at 298 K with a Bruker DRX 600-MHz spectrometer equipped with a cryogenic probe; acetone was used as an internal standard (δ_H_ = 2.225 ppm; δ_C_ = 31.45 ppm). For the proton spectrum recorded with deuterated water, the residual solvent peak was reduced by a presaturation sequence. For the proton spectrum of the pullulanase digestion product, the water peak was suppressed by applying an excitation sculpting sequence. All spectra were recorded with 16,000 points of resolution, the free induction decay was zero filled to 32,000 points, and an exponential window function (linebroadening = 0.5) was applied to enhance the signal-to-noise ratio. The spectra were processed and analyzed with Bruker TopSpin 3.1 software.

### LOS purification.

LOS was extracted as previously described (82) and examined by sodium dodecyl sulfate-polyacrylamide gel electrophoresis and silver staining as previously described (83).

### Treatment of growth medium with hyaluronidase, α-amylase, proteinase K, or hyaluronic acid.

To remove capsular material during biofilm formation, RPMI 1640 was supplemented with 0 or 300 U of hyaluronidase (Sigma-Aldrich, Raleigh, NC) prior to inoculation. In another experiment, hyaluronic acid (Sigma-Aldrich) was added to RPMI 1640 at concentrations of 0, 10, and 100 μg/ml prior to bacterial inoculation to determine the effect of extracellular CPS on biofilm formation. To assess the presence of carbohydrate or protein in the biofilm matrix, the growth medium was supplemented with 0.1 mg/ml α-amylase or proteinase K (both from Sigma-Aldrich) prior to inoculation. Biofilms were grown and analyzed as described above.

### Bacterial hydrophobicity and autoaggregation.

Bacterial hydrophobicity was determined as previously described (84), as was autoaggregation (68), with the following modifications. *P. multocida* was suspended in PBS at an OD of 1.0 at the start of the experiment. After 24 and 48 h, the OD_562_ of the top 200 μl was recorded.

### SEM.

*P. multocida* strains were grown on glass coverslips in RPMI 1640 medium without phenol red or glutamine (Lonza, Walkersville, MD) and incubated at 37°C under stationary conditions for 48 h. The coverslips were gently washed and fixed in a solution of 5% glutaraldehyde, 4.4% formaldehyde, and 2.75% picric acid in 0.05% sodium cacodylate buffer for at least 1 h. Sequential dehydration of the sample was carried out with 25, 50, 70, 80, and 95% ethanol. SEM was performed as previously described (62), with a Carl Zeiss, Inc., EVO40 scanning electron microscope.

### CLSM.

Biofilms were grown on LabTek II eight-chamber cover glass slides (Thermo Fisher Scientific, Rockford, IL) for 48 h. Biofilms were gently washed, resuspended in sterile PBS, stained with 1 μl of SYBR live stain and 1 μl of propidium iodide dead stain (Life Technologies, Inc., Frederick, MD), and incubated for approximately 1 h at room temperature. CSLM was performed with a Zeiss 880 Laser Scanning Microscope (Zeiss, Germany).

### Statistical analysis.

Median values, standard deviations, and comparative *P* values were determined by unpaired Student *t* test with Excel (Microsoft) or InStat (GraphPad Software, Inc., La Jolla, CA) software. One- and two-way analyses of variance were performed with Prism software version 6.01 (GraphPad Software, Inc.). Multiple comparisons were performed with the Sidak multiple-comparison test. Correlation of data was also performed with Prism software, version 6.01. A *P* value of ≤0.05 was considered significant. Biofilms examined by CLSM were analyzed with COMSTAT1 software (85).

### Data availability.

The sequence data obtained for the putative glycogen biosynthesis genes and proteins in this study are available at the National Center for Biotechnology Information (https://blast.ncbi.nlm.nih.gov) under protein accession numbers WP_010906715.1 (PM_RS02805), WP_005726331.1 (PM_RS02810), WP_016533562.1 (PM_RS02815), WP_005722006.1 (PM_RS02820), WP_005753940.1 (PM_RS02825), and WP_010906718.1 (PM_RS02830).
