# Erratum for Petruzzi et al., “Capsular Polysaccharide Interferes with Biofilm Formation by *Pasteurella multocida* Serogroup A”

**DOI:** 10.1128/mBio.00176-18

**Published:** 2018-02-13

**Authors:** Briana Petruzzi, Robert E. Briggs, Fred M. Tatum, W. Edward Swords, Cristina De Castro, Antonio Molinaro, Thomas J. Inzana

**Affiliations:** a Department of Biomedical Sciences and Pathobiology, Virginia-Maryland College of Veterinary Medicine, Virginia Tech, Blacksburg, Virginia, USA; b National Animal Disease Center, Agricultural Research Service, U.S. Department of Agriculture, Ames, Iowa, USA; c Department of Microbiology and Immunology, Wake Forest School of Medicine, Wake Forest University, Winston-Salem, North Carolina, USA; d Department of Agriculture, Università di Napoli Federico II, Naples, Italy; e Department of Chemical Sciences, Università di Napoli Federico II, Naples, Italy; f Virginia Tech Carilion School of Medicine, Roanoke, Virginia, USA

## ERRATUM

Volume 8, no. 6, 2017, https://doi.org/10.1128/mBio.01843-17. The name of the third author was incorrectly left off the authorship list. The byline should appear as above.

