# Supplementary material for: Capsular Polysaccharide Interferes with Biofilm Formation by Pasteurella multocida Serogroup A
Source: mBio. 2017 Nov 21;8(6):e01843-17. doi: 10.1128/mBio.01843-17 (PMC5698555; doi:10.1128/mBio.01843-17)
Supplement: TABLE S1 [file mbo006173600st1.pdf]

**Table S1. Laboratory strains and clinical isolates used in this study.** Isolates included commonly used laboratory strains and recent clinical isolates.

| Strain | Common name | Serotype[a]/ phenotype | Source |
|---|---|---|---|
| *P. multocida* subsp. *gallicida* P1059 | WT P1059 | A:3/ encapsulated | National Animal Disease Center (NADC) |
| *P. multocida* subsp. *gallicida* P1059ΔhyaE | P1059Δ*hyaE* | Non- encapsulated | This study |
| *P. multocida* subsp. *multocida* P1062 | WT P1062 | A:3/ encapsulated | NADC |
| *P. multocida* subsp. *multocida* P1062ΔhyaE | P1062Δ*hyaE* | Non- encapsulated | This study |
| *P. multocida* X73 | WT X73 | A:1/ encapsulated | NADC |
| *P. multocida* X73Δ*hyaD* | X73Δ*hyaD* | Non- encapsulated | This study |
| *Pasteurella multocida* C0513 | WT C0513 | A/ encapsulated | Experimental calf infection[3] |

| | | | |
|---|---|---|---|
| *Pasteurella multocida* C0513-P5 | C0513-P5 | A (capsule-deficient) | This study |

| Clinical Isolates | Location of Origin | Host species and infection site | Serogroup or Serotype |
|---|---|---|---|
| 756 | Virginia Department of Agriculture, Division of Animal and Food Industry Services | Chicken heart, spleen, bone marrow | F:9 |
| 765 | Virginia Department of Agriculture, Division of Animal and Food Industry Services | Turkey liver, spleen, lung | A |
| 704 | Virginia Department of Agriculture, Division of Animal and Food Industry Services | Chicken wattle | A:8 |

| | | | |
|---|---|---|---|
| 801 | Virginia Department of Agriculture, Division of Animal and Food Industry Services | Chicken heart, spleen, bone marrow | A |
| 785 | Virginia Department of Agriculture, Division of Animal and Food Industry Services | Chicken liver, spleen, heart | F:9 |
| 775 | Virginia Department of Agriculture, Division of Animal and Food Industry Services | Turkey lung | A:3,4 |
| 741 | Virginia Department of Agriculture, Division of Animal and Food Industry Services | Chicken bone marrow | A:3,4 |
| 742 | Virginia Department of Agriculture, Division of Animal and Food Industry Services | Chicken heart, spleen, bone marrow | A:1 |

| | | | |
|---|---|---|---|
| 745 | Virginia Department of Agriculture, Division of Animal and Food Industry Services | Chicken heart, spleen, bone marrow | A:3,4 |
| 747 | Virginia Department of Agriculture, Division of Animal and Food Industry Services | Chicken heart, spleen | A |
| 232 | Oklahoma State University | Bovine lung | A |
| 9877 | Oklahoma State University | Bovine lung | A |
| 96031276 | Oklahoma State University | Bovine lung | A |
| 00111378 | Oklahoma State University | Bovine nasal | A |
| 96020298 | Oklahoma State University | Bovine lung | A |
| 134 | Oklahoma State University | Bovine lung | A |
| 95101538 | Oklahoma State University | Bovine lung | A |

| | | | |
|---|---|---|---|
| 13-1367 | Virginia-Maryland College of Veterinary Medicine | Dog ear | A |
| H1 | Virginia-Maryland College of Veterinary Medicine | Cat mouth | A |
| 12B-21481 | University of Wyoming | Bovine liver, lung, small intestine | A |
| | Virginia-Maryland College of Veterinary Medicine | Porcine lung | D |
| Pm70 | ATCC (BAA-1909) | Chicken | F |
| Pm70 | Dr. Kapur, Pennsylvania State University | Chicken | F |
| 989 | Virginia-Maryland College of Veterinary Medicine | Turkey | |

[a]A standard system for designating serotypes of *P. multocida* was established using a combination of capsular serogroup typing and LPS serovar typing systems (86).
