# Supplementary material for: Capsular Polysaccharide Interferes with Biofilm Formation by Pasteurella multocida Serogroup A
Source: mBio. 2017 Nov 21;8(6):e01843-17. doi: 10.1128/mBio.01843-17 (PMC5698555; doi:10.1128/mBio.01843-17)
Supplement: FIG S1 [file mbo006173600sf1.pdf]

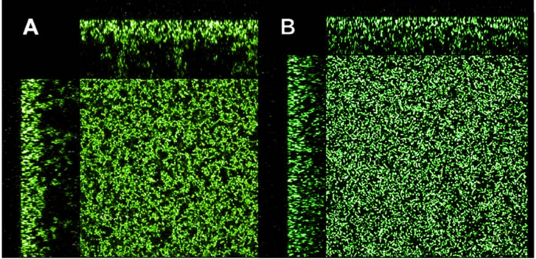

FIG S1. Cross section of the biofilm of WT P1059 biofilm (A) and P1059Δ*hyaE* biofilm (B) by CLSM. This is a cross-sectional view through the center of the biofilm shown in Fig. 6. The biofilm was stained with Syto 9. The left column and top row show the height and thickness of each biofilm, which is larger and thicker for capsule-deficient mutant P1059Δ*hyaE* than for the parent strain.
