# Supplementary material for: Capsular Polysaccharide Interferes with Biofilm Formation by Pasteurella multocida Serogroup A
Source: mBio. 2017 Nov 21;8(6):e01843-17. doi: 10.1128/mBio.01843-17 (PMC5698555; doi:10.1128/mBio.01843-17)
Supplement: TABLE S2 [file mbo006173600st2.pdf]

Table S2. Primers used for quantitative reverse transcriptase-polymerase chain reaction (qRT-PCR) analysis.

Gene description	Gene[a]	Forward primer sequence	Reverse primer sequence
**Control**	***gyrB***	**5'-TATGAGTGGCTTGGCGTTAG-3'**	**5'-ACACCGCTTCTGGATAATAGC-3'**
**Glycogen synthesis**	***malQ***	**5'-TGAACAGGCGACGAAGTATG-3'**	**5'-CCGACGAGAAGATGAGCTATAAG-3'**
**Glycogen synthesis**	***glgB***	**5'-CAGTAAAGACGGAGAGCGAATTA-3'**	**5'-CCACATAGTTACCCGCCATATT-3'**
**Glycogen synthesis**	***glgX***	**5'-AGCGATGTTTGCGCTAGAA-3'**	**5'-TCGATACCCGCTTGATGTAATG-3'**

**Glycogen synthesis**	***glgC***	**5’-GGGAACCCAGTACAAACCTATC-3’**	**5’-TAATCACGCAACCACCAGAA-3’**
**Glycogen synthesis**	***glgA***	**5’-ACATGCTTGCTCGGAGTTATAC-3’**	**5’-GGATACGCCGGTAAGACAATAC-3’**
**Glycogen synthesis**	***glgP***	**5’-CGGAATTTATCCGTCGTGTCT-3’**	**5’-GCCACGCCATTCACTTTATTC-3’**

[a]Genes selected were those present in the putative glycogen synthesis locus of *P. multocida*.
